# Artemisinin derivatives differently affect cell death of lung cancer subtypes by regulating GPX4 in patient-derived tissue cultures

**DOI:** 10.1038/s41420-025-02537-2

**Published:** 2025-05-28

**Authors:** Johanna Mölleken, Angelique Kragl, Astrid Monecke, Isabella Metelmann, Sebastian Krämer, Sonja Kallendrusch

**Affiliations:** 1 Institute of Anatomy, University of Leipzig, Leipzig, Germany; 2 Department of Thoracic Surgery, University Hospital Freiburg, Freiburg im Breisgau, Germany; 3 Institute of Clinical Research and Systems Medicine, Health and Medical University Potsdam, Potsdam, Germany; 4 Institute of Pathology, University Hospital Leipzig, Leipzig, Germany; 5 Department of Visceral, Transplantation, Thoracic and Vascular Surgery, University of Leipzig Medical Center, Leipzig, Germany

**Keywords:** Drug safety, Cancer microenvironment, Cancer therapeutic resistance, Diagnostic markers, Cancer models

## Abstract

Resistant tumor cell populations are common after cytostatic drugs treatment. To overcome resistance mechanisms artemisinin derivatives, known for its complementary use during cancer treatement and ferroptosis induction, were investigated both as single agents and in combination with cisplatin (3 µM) in a complex organotypic tissue model of non-small cell lung cancer (NSCLC) patient samples. All substances—artemisinin (ART, 100 µM), artemether (ATM, 50 µM), artesunate (ATS, 20 µM), and dihydroartemisinin (DHA, 10 µM)—showed beneficial effects in most of the investigated patient-derived tissue cultures (PDTC). Tumor proliferation was reduced by DHA and ATS in both, standalone treatment and in combination with cisplatin, surpassing the efficacy of single cisplatin supplementation. In combination with cisplatin tumor apoptosis increased in most of lung squamous cell carcinoma (LUSC) PDTC, but not in lung adenocarcinoma (LUAD). The enzyme GPX4, inhibiting ferroptosis was up-regulated in LUAD but not in LUSC. Taken together, in the complex PDTC model system, LUSC displayed a higher sensitivity to ART derivatives, due to the lack of GPX4-mediated resistance to ferroptosis.

## Introduction

Tumor mutation rates lead to a fast adaptation of tumor cells to tissue changes and consequently resistance mechanisms [1]. To overcome resistance of tumor cell populations novel or complementary approaches are needed to enhance the effectivity of clinical established drugs. Artemisinin (ART), a herbal drug from Traditional Chinese medicine, and its semisynthetic derivatives Dihydroartemsinin (DHA), Artesunate (ATS) and Artemether (ATM) are an inherent part of the treatment of *Plasmodium falciparum* malaria [2]. A variety of studies investigated the anticancer impacts of ART and its derivatives in both, in vivo and in vitro as well as in multiple tumor entities [3–8] such as non-small-cell-lung cancer (NSCLC) [9–11]. They discern in water solubility, lipophilicity, half-life and bioavailability, whereas ART and its active metabolite DHA have the highest bioavailability [12]. Thereby, these compounds interfere with different pathways and can cause apoptosis, ferroptosis and cell cycle arrest as well as inhibition of proliferation, metastasis and angiogenesis of cancer cells. However, these results were mostly obtained from analysies of single tumor cell lines [9, 13–15]. Still, the plethora of mechanisms addressed by artemisinin and its derivatives also do involve the tumor microenvironment (TME). Tumor heterogeneity and TME have a great importance [16] for applicability to clinical practice and therefore to understand tumor response to different options of treatment. Therefore, it is a challenge to find a model that reflects a similar situation as in the individual patient. Patient-derived slice cultures (PDTC) are established for numerous tumor entities and besides clinical relevance, the maintenance of the TME heterogeneity makes this model system suitable to investigate complex resistance patterns [17–20].

The analysis of 188 lung cancer cases identified 1013 mutations in the lung cancer genome [21, 22]. The distribution of the genes across cellular pathways was spread over five pathways, demonstrating once more the heterogeneity of lung cancer. Lung cancer causes the majority of cancer-related deaths worldwide and has a stable incidence of approx. 2.2 million per year [23, 24]. NSCLC is accounting for approximately 85% of all lung cancer. Further, NSCLC is barely a homogenous entity, but a group that discerns in clinical, morphological and biomolecular patterns. It is classified in two major groups, apart from a lot of minor groups: squamous cell carcinoma (LUSC) and adenocarcinoma (LUAD) [25, 26]. The treatment was enhanced and specified in the last 30 years and consists of various prospects such as surgery, chemotherapy, immune checkpoint inhibitors and radiation, many a time in combination [25]. Despite that, overall survival of patients has only slightly improved. Therefore, cisplatin is even after more than 40 years among the most widely used drug in lung cancer treatment. Once cisplatin enters the cell it exerts its cytotoxic effect by losing one chloride ligand, binding to DNA to form intra-strand DNA adducts, inhibiting DNA synthesis and cell growth. The DNA lesions formed from cisplatin-induced DNA damage activate DNA repair responses, consequently leading to resistant tumor cell populations. Therefore, many patients experience relapse due to drug resistance and combination therapies using platinum-derived drugs and immune checkpoint inhibitors have become the preferred approach [27, 28]. For example, the immune checkpoint PD-1 or PD-L1 can be blocked by an antibody to enhance immune activation. However, long-term responses remain rare, and resistance frequently occurs [29]. Consequently, herbal therapies are more and more adapted by patients, with or without the knowledge of the treating practitioner [30], without robust evidence of effectivity in combinatory approaches.

Here, we investigated artemisinin, dihydroartemisinin, artesunate and artemether in the patient-derived system model of NSCLC. No adverse effects of combined administration of clinical relevant doses of cisplatin and the active compound DHA was seen. However, LUAD cells of PDTC, but not LUSC cells were resistant to ferroptosis by enhancing GPX4 expression.

## Results

### Effects of artemisinin and its derivatives, DHA, ATS and ATM on tumor cell survival in PDTC of NSCLC

**Fig. 1:**
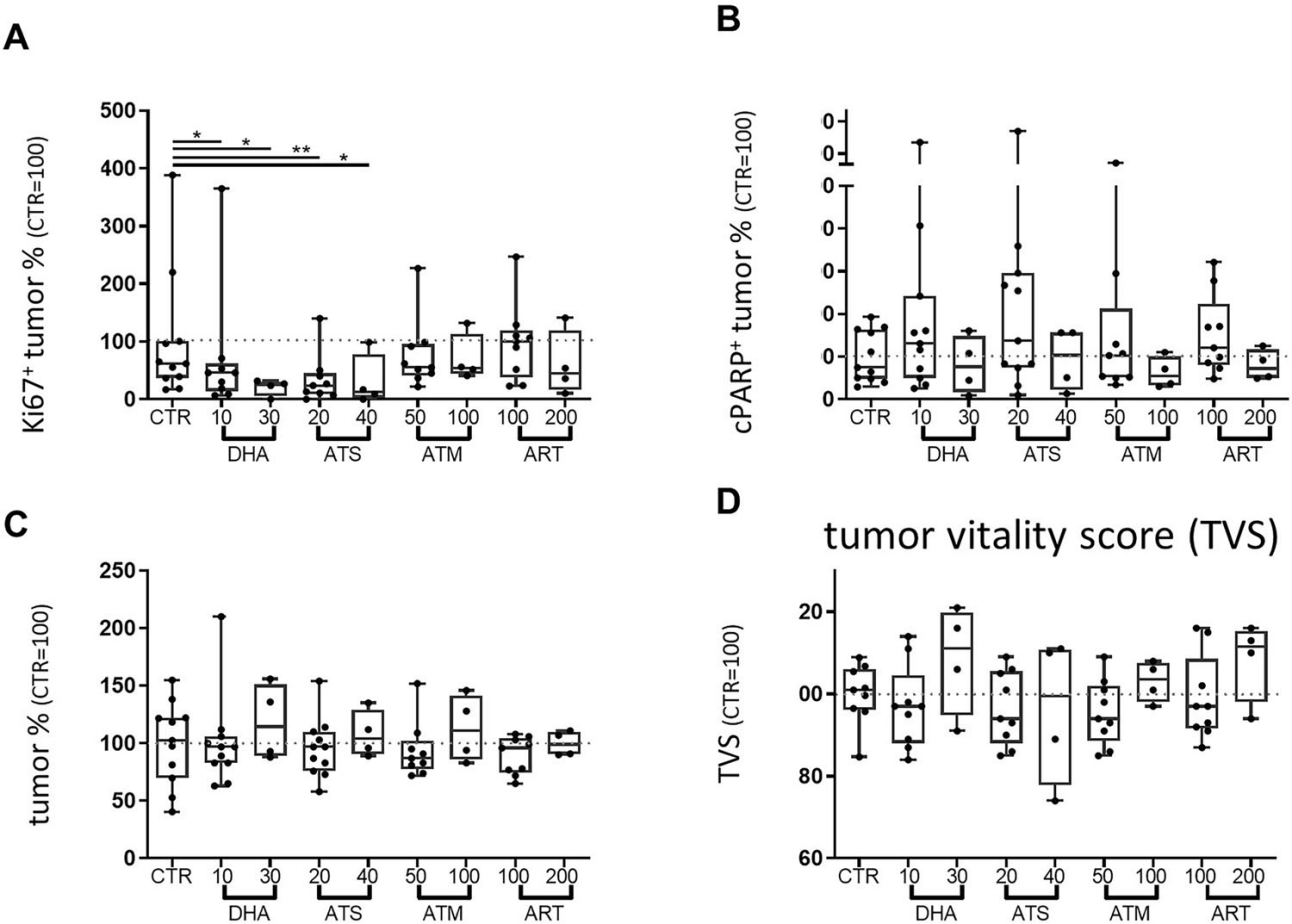
Effects of artemisinin and its derivatives DHA, ATS and ATM in PDTC of NSCLC. Up to twelve PDTC, treated for 72 h are shown. The tumor proliferation (TP) demonstrate a reduced expression of Ki67 in tumor cells after DHA and ATS treatment independent of the concentration used (**A**). Tumor apoptosis (TA), determined by cParp, does show enhanced cPARP expression in some PDTC and only in low-dose applications (**B**). Effects on tumor fraction (TF) (**C**). All parameters, TA, TP and TF were combined to one final score, determining the tumor vitality (TVS, **D**) The percentage of the respective control (100%) is given, to demonstrate inter-tissue variability control variances are indicated in the CTR. The median of each condition is indicated. (*N* ≤ 12, Kruskal–Willis test with Dunn´s correction *p* < 0.05 vs. CTR).

All substances were tested in PDTC from surgical resections with two different concentrations over 72 h. All evaluated PDTC (*n* = 4–11) maintained their individual tumor fraction ex vivo (variation coefficient of CTR 35.81%). While there was no significant alteration in the tumor fraction, median ranging from 87% (ART 100 µM) to 114% (DHA 30 µM), a significant decrease in tumor proliferation was observed with DHA (median: 46%,10 µM (p < 0.05) and 25%, 30 µM (*p* < 0.05)) and ATS (median: 24%, 20 µM (*p* < 0.005) and 13%, 40 µM (*p* < 0.05)) whereas ART (55%, 100 µM and 54%, 200 µM) and ATM (100%, 50 µM and 45%, 100 µM) did not reach significance (Fig. 1A). No significant alteration was also observable in the tumor apoptotic cell fraction, due to high variances of the individual PDTC (variance coefficient range: 45–106%). Median values of the lower concentrations (*n* = 9–11) showed an elevated trend in comparison to the control condition (DHA: 131%,10 µM; ATS: 137%, 20 µM; ART: 103%, 100 µM; ATM: 121%, 50 µM) while the higher concentrations (*n* = 4) do not show elevated fractions of tumor apoptosis (DHA: 77%,30 µM; ATS: 104%, 40 µM; ART: 54%, 200 µM; ATM: 72%, 100 µM Fig. 1B). Due to the observed extensive range in the response to treatment of artemisinin and its derivatives, the results were further evaluated. To this end, a tumor vitality scoring of all three parameters (TF, TP and TA) was used to categorize response of each individual PDTC reaction. A population of patient samples that was more susceptible to artemisinin and its derivatives was observed by using the tumor vitality score with lower doses showing a beneficial trend (Fig. 1C, D).

**Fig. 2:**
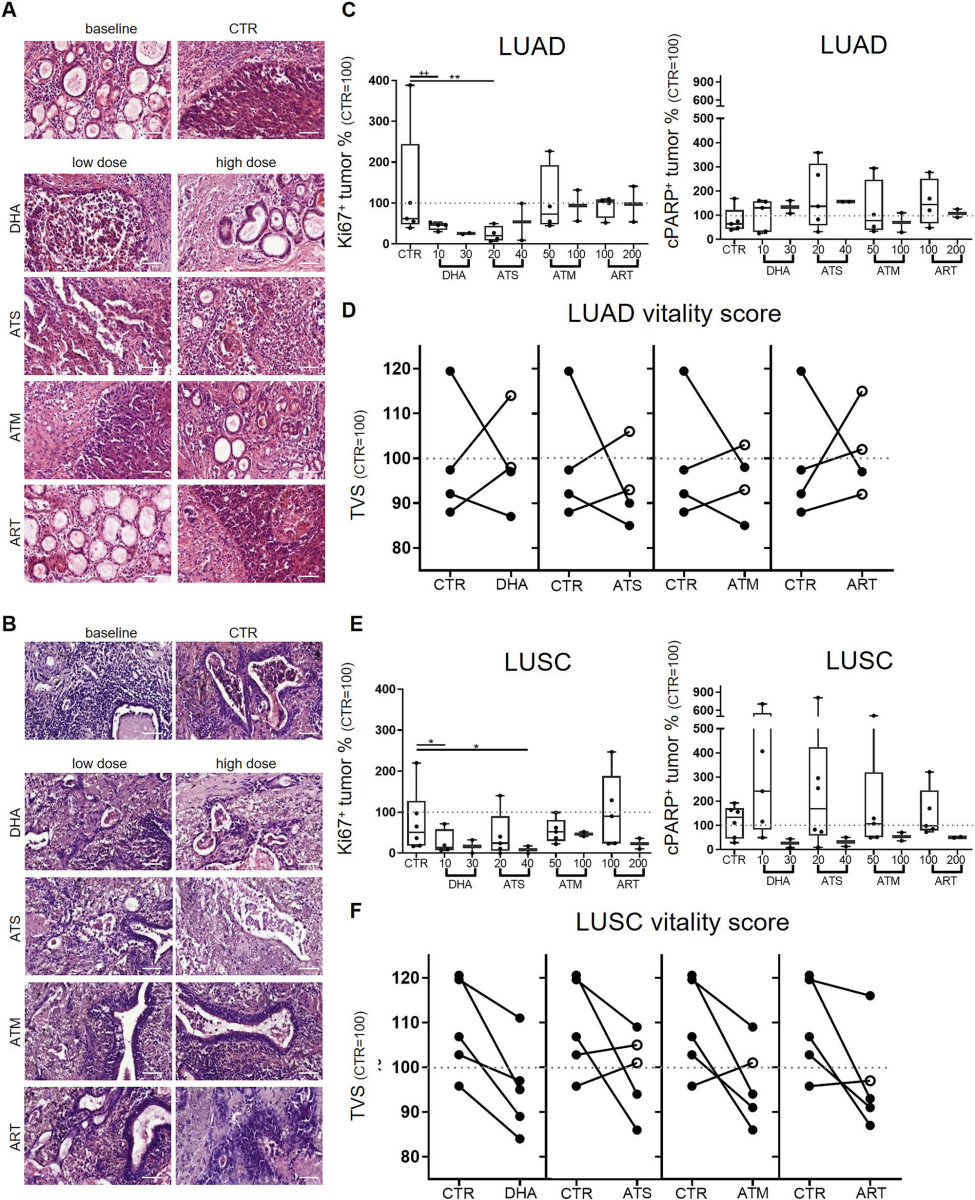
Differential effects of artemsinin and its derivatives in LUAD and LUSC. HE staining reveals differential effectivity. Shown are representative pictures of baseline (native tissue) the culture control (CTR) and each treatment performed of one patient with adenocarcinoma (#16, Bars = 100 µm, **A**) and one patient with squamous cell carcinoma (#18, Bars = 100 µm, **B**). LUAD proliferation under treatment does decrease after DHA and ATS supplementation, but the cParp expression does not alter (*N* = 5, **C**). The tumor vitality score demonstrate that two to three PDTC do not obtain benefit of the treatment (**D**). LUSC proliferation under treatment does decrease after DHA and ATS supplementation, although not significant, cParp expression was elevated in some PDTC. (*N* = 6, **E**). The tumor vitality score demonstrate that all PDTC reduce the tumor vitality under DHA treatment (**F**). The percentage of the respective control (100%) is given, to demonstrate inter-tissue variability control variances are shown. The median of each condition is indicated. (^++^Mann–Whitney U-test, *p* < 0.005 vs. CTR, **Kruskal–Willis test with Dunn´s correction *p* < 0.005 vs. CTR).

High tissue heterogeneity was observable in H&E-stained PDTC of LUAD (Fig. 2A) and LUSC (Fig. 2B). The tissue itself undergoes minor adaptations under cultural conditions, as previously shown [19]. All derivatives effected the tumor cells and demonstrated a more pronounced effectivity of DHA and ATS. Still, considering the different origin of the tumor cells, higher responsiveness of LUSC PDTC was suggested.

### Differential effects of artemisinin and its derivatives in LUAD and LUSC

Consideration of the two major subtypes in NSCLC revealed a similar effectivity of DHA (median LUAD: 46% (10 µM, *p* < 0.05) and 26% (30 µM); LUSC: 14% (10 µM, *p* < 0.05) and 16% (30 µM)) and ATS (median LUAD: 20% (20 µM, *p* < 0.005) and 54% (40 µM); LUSC: 24% (10 µM, *p* = 0.037) and 8.5% (40 µM, *p* < 0.05)) to reduce tumor cell proliferation. Supplemented in higher concentrations, ART and ATM showed less effects on the tumor proliferating cell fraction in PDTC of LUAD (median ART: 74% (100 µM) and 94% (200 µM); ATM: 103% (50 µM) and 98% (100 µM)) than in PDTC of LUSC (median ART: 52% (100 µM) and 47% (200 µM); ATM: 90% (50 µM) and 23% (100 µM)) (Fig. 2C, E). In contrast, no significant effects were observed examining the tumor apoptotic cell fraction in LUAD (median, DHA: (131% (10 µM), 135% (30 µM); ATS: 137% (20 µM), 156% (40 µM); ART: 79% (100 µM), 70% (200 µM); ATM: 145% (50 µM), 109% (100 µM)) and LUSC (median, DHA: (242% (10 µM), 27% (30 µM); ATS: 169% (20 µM), 32% (40 µM); ART: 107% (100 µM), 54% (200 µM); ATM: 99% (50 µM), 51% (100 µM)) (Fig. 2C, E). Although tumor cell apoptosis did not reach statistical significance, three PDTC did show robust alterations when supplemented with DHA and ATS. Two of these three patient samples were treated with ART and ATM, and showed a higher apoptotic tumor fraction than in their respective control conditions. The tumor vitality score, combining tumor- fraction, -apoptosis and -proliferation visualizes the effect of the applied drug on each patient sample in direct correlation with its respective control (Fig. 2D, F). While in LUAD two to three PDTC did not benefit from the applied derivatives all PDTC of LUAC had a decreased tumor vitality score when DHA (10 µM) was applied. No beneficial effect was observable in one PDTC of LUSC when ATS, ART or ATM was applied and another PDTC showed no reactivity to ATS. The tumor fraction parameter as well as representative immunfluorescent stainings are shown in Supplementary Fig. 1.

### Complementary effects of dihydroartemisinin and artesunate with cisplatin in LUAD and LUSC

**Fig. 3:**
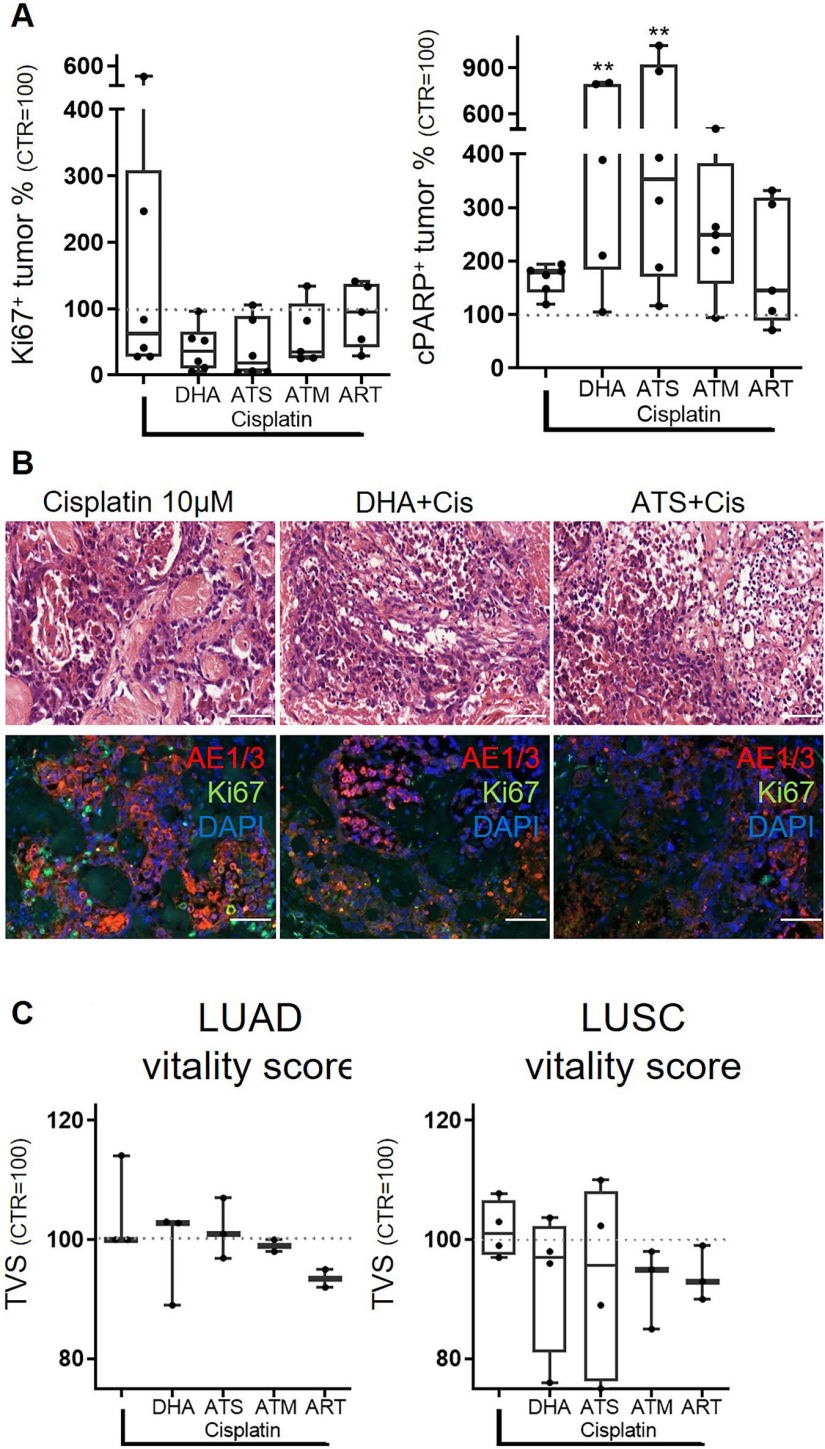
Combination of cisplatin and derivatives of artemisinin demonstrate beneficial effects. The large variance of tumor proliferation upon cisplatin is diminished by artemisinin derivatives, while tumor apoptosis increases after combinatory administration of cisplatin and DHA (*N* = 6, **A**). Representative pictures are shown, demonstrating the massive increase of apoptotic cell bodies in HE and the reduction of Ki67 positive staining in combined treatment conditions. (Bars = 50 µm, **B**). Tumor fraction (TF), tumor apoptosis (TA), and proliferation (TP) were combined into one final tumor vitality score (TVS), showing the efficacy of the combinatory treatment against cisplatin in LUSC (LUAD: *N* = 3, LUSC: *N* = 4, **C**) The median of each condition is indicated. (^++^Mann-Whitney *U*-test, *p* < 0.005 vs. CTR).

To analyze complementary effects, single treatment with cisplatin (3 µM, median: tumor proliferation 62% vs. CTR, tumor apoptosis 178% vs CTR)) was combined with either DHA (10 µM), ATS (20 µM), ART (100 µM) or ATM (50 µM). Tumor proliferation and tumor apoptosis were significantly altered by combined treatment wiht DHA and cisplatin (TP: 36% (*p* < 0.05 vs. CTR), TA: 421% (*p* < 0.005)) as well as ATS and cisplatin (TP: 17% (*p* < 0.05 vs. CTR), TA: 353% (*p* < 0.005 vs. CTR) Fig. 3A, B). ATM in combination with cisplatin did enhance median TA to 249% and reduced TP to 35%, also showing a stronger effect on tumor vitality than single cisplatin treatment. Applying the tumor vitality scoring, subtype-specific differences in treatment efficacy were observed (Fig. 3C). In LUSC, an overall reduced tumor vitality was detected. In LUAD, beneficial effects were only detected when ART in combination with cisplatin was applied (mean: 94% vs. 100% cisplatin).

### Complementary effects of dihydroartemisinin and artesunate with PD-1 inhibition in LUAD and LUSC

**Fig. 4:**
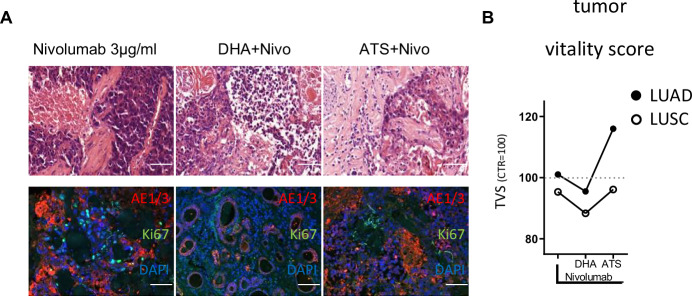
Case reports on combination of nivolumab and derivatives of artemisinin demonstrate beneficial effect in LUSC. Representative pictures are shown, demonstrating the massive increase of apoptotic cell bodies in HE and the reduction of Ki67 positive staining in combined treatment conditions of the investigated LUSC case. (Bars = 50 µm, **A**). Tumor fraction (TF), tumor apoptosis (TA), and proliferation (TP) were combined into one final tumor vitality score (TVS), showing the efficacy of the combinatory treatment with DHA against nivolumab in LUSC (LUAD: N = 1, LUSC: N = 1, **B**).

One PDTC of LUSC and one PDTC of LUAD was treated in combination with nivolumab. While DHA could demonstrate a reduced tumor vitality score in LUSC (88%), no beneficial effect was observed in LUAD (96%, Fig. 4A). ATS did not alter the tumor vitality score in LUSC (96%) and showed adverse effects in LUAD (116%, Fig. 4B). Single parameters of the shown tumor vitality scoring are given in Supplementary Fig. 2.

### Depressed GPX4 expression promotes higher susceptibility of LUSC to artemisinin and its derivatives

As DHA and ATS showed a robust regulation of TA and TP, these conditions were selected for further analysis, investigating the effect of ferroptosis regulation in PDTCs of NSCLC. First, ferritin expression and transferrin-receptor expression were explored to investigate their regulation after enhanced tumor apoptosis. Although cisplatin could alter ferritin expression (LUAD, 140%; LUSC, 54%) no effect of DHA an ATS could be measured (Supplementary Fig. 3). To elucidate the effect of DHA and ATS on tumor-associated macrophages, staining with the CD68 antibody was conducted. Although a diminished CD68 positive macrophage population might indicate some influence, no further treatment confirmed this observation. The enhanced reactivity of macrophages was mostly induced by the massive cell-death induced by cisplatin (Supplementary Fig. 3).

**Fig. 5:**
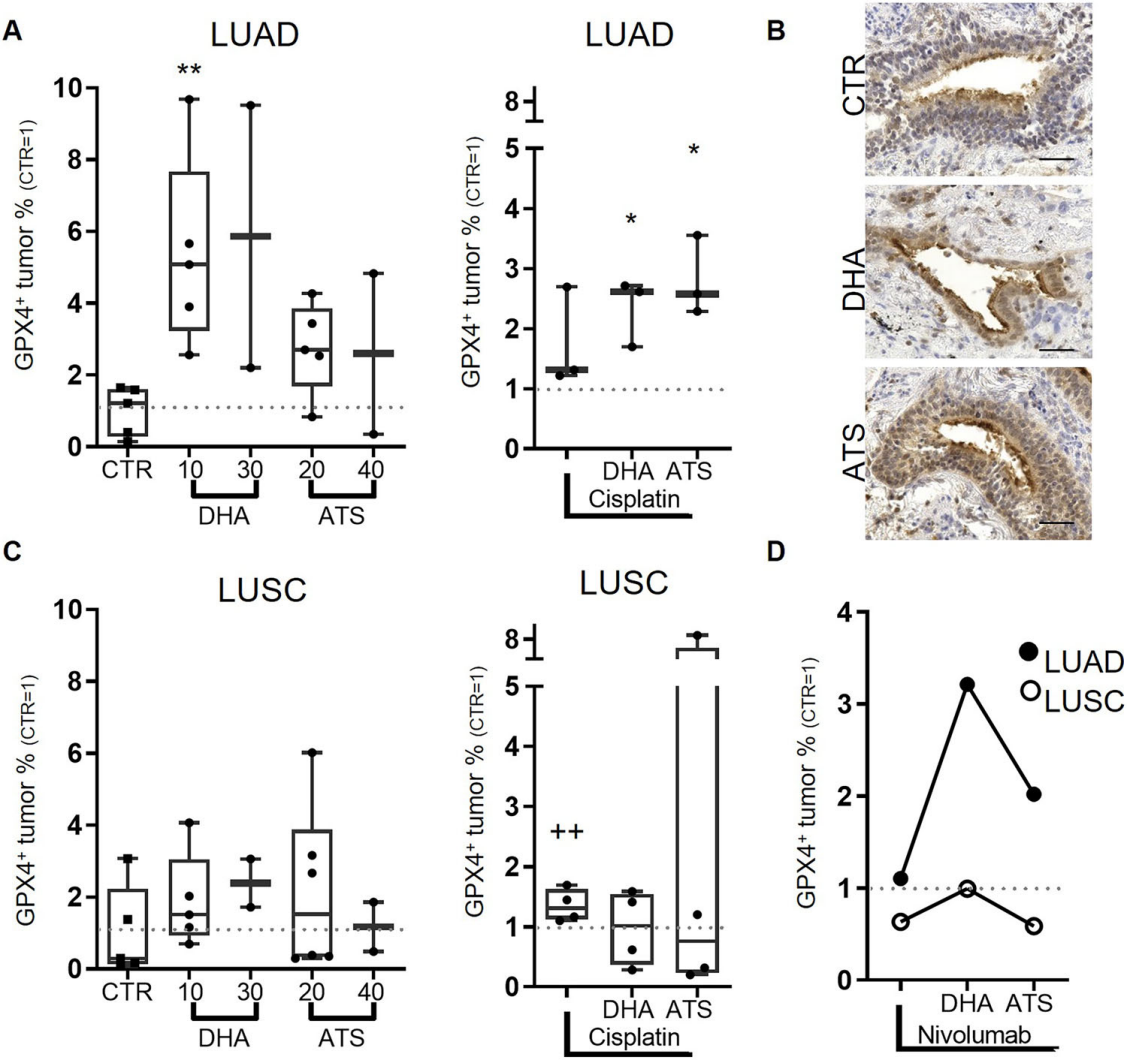
GPX4 expression is enhanced by DHA in LUAD but not in LUSC. In LUAD DHA treatment provoked an increase in GPX4 expression, which was also observed in combinatory treatment with cisplatin (LUAD: *N* = 5 and 3, **A**). Representative pictures of GPX4 expression. (Bars = 50 µm, **B**). In LUSC all treatment conditions do not show an alteration in GPX4 expression (LUSC: *N* = 5 and 4, **C**). The GPX4 expression in the combinatory approach with nivolumab was high in the PDTC of LUAD but remained low in PDTC of LUSC (LUAD: *N* = 1, LUSC: *N* = 1, **D**) The percentage of the respective control (100%) is given, to demonstrate inter-tissue variability control variances are shown. The median of each condition is indicated. (*Kruskal-Willis test with Dunn´s correction *p* < 0.05 vs. CTR, ^+^Mann-Whitney *U*-test, *p* < 0.05 vs. CTR).

A high heterogeneity of intertumoral GPX4 regulation was observed in all investigated PDTCs under treatment with single derivatives and cisplatin treatment (Supplementary Fig. 4). GPX4 expression was increased by single DHA (median: 256% (10 µM, *p* < 0.05 vs. CTR)) and single cisplatin (median: 132%, *p* < 0.05 vs. CTR)) treatment. Discriminating LUAD and LUSC, a significant increase of GPX4 in LUAD under DHA treatment (median: 508% (10 µM, *p* < 0.005 vs. CTR)) as well as a clear trend towards enhanced GPX4 expression was observed with ATS (median: 270% (20 µM)). In combination with cisplatin this effect was still observable in both applied derivatives in PDTC of LUAD (median: Cis + DHA: 261% (*p* < 0.05 vs. CTR); Cis+ATS: 258% (*p* < 0.05 vs. CTR)). Single cisplatin administration significantly altered GPX4 expression only in LUSC, which may be caused by the higher sample size and less variance (LUSC median: 131%, *t*-test *p* < 0.005, LUAD median: 132% Fig. 5A, B). However, no up-regulation of GPX4 was observed in PDTC of LUSC treated with DHA (median: 134%) and ATS (median: 153%). In combination with cisplatin GPX4 expression remained further unchanged in PDTC of LUSC (median: Cis + DHA 102%; Cis+ATS 76%, Fig. 5C).

In combination with nivolumab, DHA and ATS altered GPX4 expression in one PDTC of LUAD (median: DHA+nivolumab 321%, ATS+nivolumab 202%) but not in LUSC (median: DHA+nivolumab 99%, ATS+nivolumab 59%, Fig. 5D).

## Discussion

The ferroptosis inhibitor GPX4 showed subtype-dependent regulation in PDTC of NSCLC ex vivo correlating with the tumor apoptotic fraction. This effect was even more pronounced in combination with cisplatin, an integral part of most adjuvant regimens [31].

The nobel price in medicine was dedicated to Youyou Tu for the discovery of artemisinin, found through a recipe described by Gong He approx. 1700 years ago. Since then, artemisinin has been shown to be pharmacologically relevant, mainly for antimalarial but also anticancer activities [32–34]. Still, its bioavailability with a short half-life and poor water solubility range in the micromolar or higher nanomolar concentrations after long-term oral administration of ATS. This was shown in plasma and in lung tissue of rats [35–38]. Nevertheless, nano-targeted drug delivery systems to overcome this limitation and improve anti-tumor efficacy is discussed elsewhere [39].

In the present study, the applied concentrations correlate with concentrations, mostly used in cell culture experiments and did only consider relevant doses of DHA, the active metabolite [40]. We investigated also the effect of ATM and ATS, both regarded as prodrug in malaria treatment. However, the methyl ether derivative of DHA, ATM, is an oil-soluble derivative with more hydrophobic properties [41] and was therefore investigated in PDTC. To estimate an overall drug effect tumor proliferation, apoptosis and the tumor fraction of each PDTC was determined and combined to a tumor vitality score, as reported previously [42]. Surprisingly, ATM as well as DHA and ATS showed beneficial effects, reducing the total tumor fraction of PDTC. These effects are similar to effects observed with immune checkpoint-inhibitors [19, 42] and is supported by the wide range of immunoregulatory functions of artemisinin derivatives in immune-mediated inflammatory diseases [43]. In tumor-bearing mice, suppression of tumor tissue was observed and one study also reported T-reg depletion following ATM administration [41, 44, 45].

Even though, the combination of DHA and ATS with an immune-checkpoint inhibitor against PD-1, was investigated only in two PDTC, a beneficial effect was observable in LUSC. However, PDTC from the patient with LUAD did not show beneficial alterations and even suggests underlying resistance development. Further research, addressing the immunological properties of ART and its derivatives in PDTC could provide insights of underlying short-term mechanisms.

Investigating tumor proliferation, only ATS and DHA showed a stable decrease in all NSCLC-derived PDTC. Only one clinical trial was conducted so far in lung cancer and could demonstrate a prolonged time to progression when vinorelbine and cisplatin were administered with ATS (120 mg) IV for eight days, proving safety an no adverse effects [38]. The less potent derivatives ATM and ART altered tumor proliferation only in samples obtained from LUSC patients. The anti-proliferating properties of the investigated drugs were attributed to the known anti-proliferative mechanisms of cell cycle regulation, Wnt/β-catenin pathway inhibition, that were demonstrated in many preclinical models of various tumor entities, and was not further investigated in the present study [13, 46, 47].

Still, further pathways, inhibiting cell proliferation are caused by reactive oxygen species-dependent DNA damage or autophagosome formation [47, 48]. These mechanisms are closely related to cell death and were investigated as the apoptotic potential of DHA and ATS was only present in PDTC of LUSC tissue samples. Only when DHA and ATS were administered together with cisplatin the apoptotic tumor fraction of LUAD were elevated. This effect however, was relevant even in comparison to cisplatin.

As ferroptosis and autophagy are interconnected through the regulation of cellular iron homeostasis and cellular ROS, we investigated ferritin expression as well as the transferrin receptor expression to assess whether cells regulate their iron metabolism in response to derivate treatment. Neither ferritin, nor the immunohistochemical analysis of the transferrin receptor 1 expression did show regulatory effects after DHA or ATS supplementation. In a cell line of a human LUAD, the mRNA level of transferrin receptor was upregulated by ART and DHA [14]. This effect could not be confirmed in the complex system model of PDTC. Still, cisplatin showed reduced ferritin expressions, which were restored in combination with DHA and ATS to their respective control conditions. This suggests that autophagy is increased by cisplatin and might be restored by DHA and ATS. We could only observe this effect in PDTC of LUSC. As ferritin degradation leads to increased iron levels, resulting in oxidative stress by the Fenton reaction, ferroptosis should be increased, leading to enhanced death of tumor cells [49]. Previous studies also demonstrated that ATS induced ferritinophagy and consequently ferroptosis by up-regulating the nuclear receptor coactivator 4, a selective cargo receptor for the turnover of ferritin [50–52]. To determine that the observed effects of DHA and ATS were related to ferroptosis, we studied the expression of glutathione peroxidase (GPX4), an essential regulator of ferroptosis [53, 54]. No alteration of GPX4 expression took place in all investigated PDTC of LUSC, neither after single DHA or ATS administration or combined administration with cisplatin or PD-1 inhibition. On the other side, GPX4 was upregulated in PDTC of LUAD after single administration of DHA and ATS and combined administration with cisplatin and nivolumab. It was previously shown that inhibition of GPX4 can induce the death of cancer cells resistant to conventional chemotherapy [55]. Cisplatin did not show major alteration in the investigated PDTC and ART and its derivatives induced cell death of tumor cells in combined application. This effect cannot be related to GPX4 regulation alone, although resistant cancer cell were previously shown to become vulnerable upon ferroptosis inducers [56]. Many more potential pathways of ART and its derivatives are discussed in the literature, which could not be addressed in the present study [57–59]. The immunohistochemical staining of CD68 macrophages only allowed for the determination of very obvious alterations in the regulation of this immune cell population. However, as evidence continues to accumulate demonstrating the immune-regulating effects of DHA [60–62], the present model system, which preserves relevant immunological properties, should be further utilized to explore its mechanisms of action and potential resistance pathways [18, 19, 60].

Although this study demonstrates the powerful effect of ART and its derivatives in NSCLC, adverse effects demonstrated in single case reports, need to be careful considered investigated prior clinical implementation, especially in combined therapeutic approaches [63]. As the model system of PDTC preserves the tissue on a liquid-air interface and maintains an organotypic cellular composition and architecture, a more realistic estimation of the mode of action in cancerous tissue can be made. The shown difference of GPX4 regulation in LUAD and LUSC needs to be further investigated, to explore its significance for clinical therapeutic interventions.

## Materials and methods

### Specimen

Tumor samples were collected from patients treated at university hospital in Leipzig, Germany. Overall, 12 patients suffering NSCLC were included. Baseline characteristics of the patients and their disease are summarized in Table 1. The study was approved by the Ethics Committee of the Medical Faculty, University of Leipzig (Protocol No. 370-1316122013). All patients declared their written informed consent to this study and all methods were performed in accordance with the relevant guidelines and regulations.

**Table 1:**
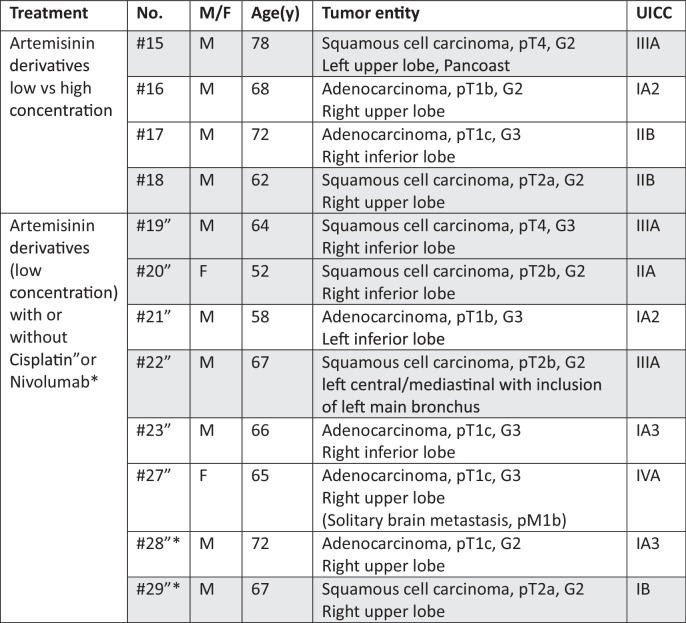
Clinical tumor sample data.

grey = LUSC, white = LUAD.

### Preparation of tissue slice cultures

The preparation protocol was previously described [19] and applied with some modifications. Fresh tumor tissue was obtained immediately after surgical resection and pathological assessment. Samples were cut into slices of 350 µm using a tissue chopper (McIlwain TC752; Campden Instruments, Leicestershire, England), followed by standardisation of 3 mm diameter with a coring tool (kai Europe, Solingen, Germany). Three tissue slices were selected randomly, placed on membrane inserts (Millipore Corporation, Billerica, USA) and cultivated in six-well plates under standardized conditions of 37 °C and 5% CO_2_. Medium was changed after 24, 48 and 72 h. Eventually the treated tumor tissue and untreated controls were fixed after 96 h of cultivation with 4% paraformaldehyde (PFA) overnight. Baseline samples were fixed on day of preparation.

### Experimental setup and treatment

All substances were tested on PDTCs from surgical resections with two different concentrations over 72 h. Artemisinin (ART) and its semisynthetic derivatives Dihydroartemisinin (DHA), Artesunate (ATS) and Artemether (ATM) were purchased from Sigma-Aldrich (St Louis, Missouri, USA) and soluted in DMSO (AppliChem, Darmstadt, Germany). The stocks were aliquoted and stored at -80 °C. Shortly before treatment, aliquots were defrosted and diluted in medium (phenol-free RPMI 1640 (Thermo Fisher scientific, Waltham, USA), which was supplemented with 1% penicillin/streptomycin (Merck, Darmstadt, Germany; 10 000 U penicillin/10 mg/ml streptomycin in 0.9% NaCl), 1% l-glutamine (Thermo Fisher Scientific, 200 mM) and 10% fetal calf serum (Thermo Fisher Scientific). Four Specimen (#15, #16, #17, #18) were treated with a low and a high concentration which distinguished between the derivatives caused by their different bioavailability. DHA was applied in 10 µM and 30 µM, ATS in 20 µM and 40 µM, ATM in 50 µM and 100 µM and ART in 100 µM and 200 µM.

Six Specimen (#19, #20, #21, #22, #23, #27) were treated with low concentration of the derivatives as well as in combination with Cisplatin 10 µM (medac GmbH, Germany). Two Specimen (#28, #29) were treated with DHA or ATS in low concentration, respectively combined with Cisplatin 3 µM or Nivolumab 3 µg/ml (medac GmbH, Germany). Considering the cytotoxic effect of DMSO, control conditions with 0,1% were performed in each experimental setup.

### Staining procedure

PFA (4%) fixed slices were embedded in paraffine and processed to 7 µm sections. Haematoxylin and eosin (H&E) staining was performed to characterize histopathological aspects and evaluate tumor cell proportion. Proliferating tumor cell section, apoptotic tumor cell fraction, cell count and tumor cell count were analysed by immunofluorescence staining. Expression of CD68 and CD71/transferrin receptor 1 were investigated in immunohistochemical staining. Slices were deparaffinized, washed with 0.3% phosphate buffered saline/Triton X and blocked for 30 minutes with Normal Goat Serum (Jackson ImmunoResearch, Ely, Cambridgeshire, UK). Primary antibodies against cleaved PARP (abcam, Cambridge, UK, rabbit, 1:100), Ki67 (DCS, Hamburg, Germany, rabbit, 1:200), cytokeratins AE1/3 (BioGenex, Fremont, CA, USA and Dako, mouse, 1:100), Ferritin (Sigma-Aldrich, St. Louis, USA, rabbit, 1:800), GPX4 (abcam, ab125066, 1:500), CD68 (PG-M1, Dako, mouse, 1:500) and CD71/transferrin receptor 1 (H68.4, Thermo Fisher, mouse, 1:250) were soluted in 0.5% bovine serum albumin and incubated overnight at 4 °C. Sections were washed with PBS/TritonX and second antibodies were applied for 1h at room temperature. For immunofluorescence staining, we used goat anti rabbit 647 and goat anti mouse 568 (AlexaFluor, Invitrogen; Eugene, OR, USA, 1:500 respectively 1:250). Slices stained with diaminobenzidine (DAB, Sigma-Aldrich) were counterstained with haematoxylin. For second antibody we used biotinylated goat anti-mouse (Sigma-Aldrich, 1:100). Nuclei were stained with DAPI (4′,6-Diamidin-2-phenylindol) or Hoechst 33342 (Sigma-Aldrich).

### Analysis

HE stained sliced were investigated using slide-scans (Panoramic SCAN and Panoramic Viewer, 3D Histech, Budapest, Hungary). Tumor specimen with extensive fibrosis and lack of viable tumor cells in baseline or control conditions were excluded from further Analysis.

From fluorescence-stained tissue, 3 pictures (x20) per slice were manually taken with an Olympus BX51 fluorescent microscope (Olympus Deutschland, Hamburg, Germany). We used a semi-automatic pixel counting algorithm for Image J Fiji [19]. Positive Pixels of Hoechst 33 342, respectively DAPI, AE1/3, cleaved PARP, Ki67 and Ferritin were counted to quantify tumor cell fraction (Hoechst- and AE1/3 positive pixels), proliferating tumor cell fraction (Hoechst-, AE1/3- and Ki67-positive pixels) apoptotic tumor cell fraction (Hoechst-, AE1/3- and cleaved PARP positive pixels) and ferritin as well as GPX4 expressing tumor cell fraction (Hoechst-, AE1/3- and Ferritin positive pixels). We normalized each condition to their respective control condition with culture media or DMSO (CTR, DMSO = 1). Effects on tumor fraction (TF), tumor apoptosis (TA), and proliferation (TP) were combined into one final tumor vitality score (TVS; 1 + TF − (1.2 ∗ TA) + (0.8 ∗ TP)) as applied previously to assess drug efficacy [42]. For statistical analysis, mean values for conditions were calculated using mean slice values and standard error of the mean (SEM). For combination of different experiments, mean values and SEM were calculated from mean condition values. GraphPad Prism 8 (GraphPad software) was used for calculating non-parametric one-way analysis of variance with Dunn’s post-test correction.

## Supplementary information

Subtype dependent tumor fraction of all PDTC samples

Effects of cisplatin and nivolumab with artemisinin and its derivatives DHA, ATS and ATM in PDTC in NSCLC

Ferritin expression is not regulated by DHA and ATS in PDTC of NSCLC

GPX4 expression in PDTC of NSCLC

## Data Availability

The datasets analyzed during the current study are available from the corresponding author on reasonable request.
