# Supplementary figures and images for: Artemisinin derivatives differently affect cell death of lung cancer subtypes by regulating GPX4 in patient-derived tissue cultures
Source: Cell Death Discov. 2025 May 28;11:256. doi: 10.1038/s41420-025-02537-2 (PMC12119945; doi:10.1038/s41420-025-02537-2)

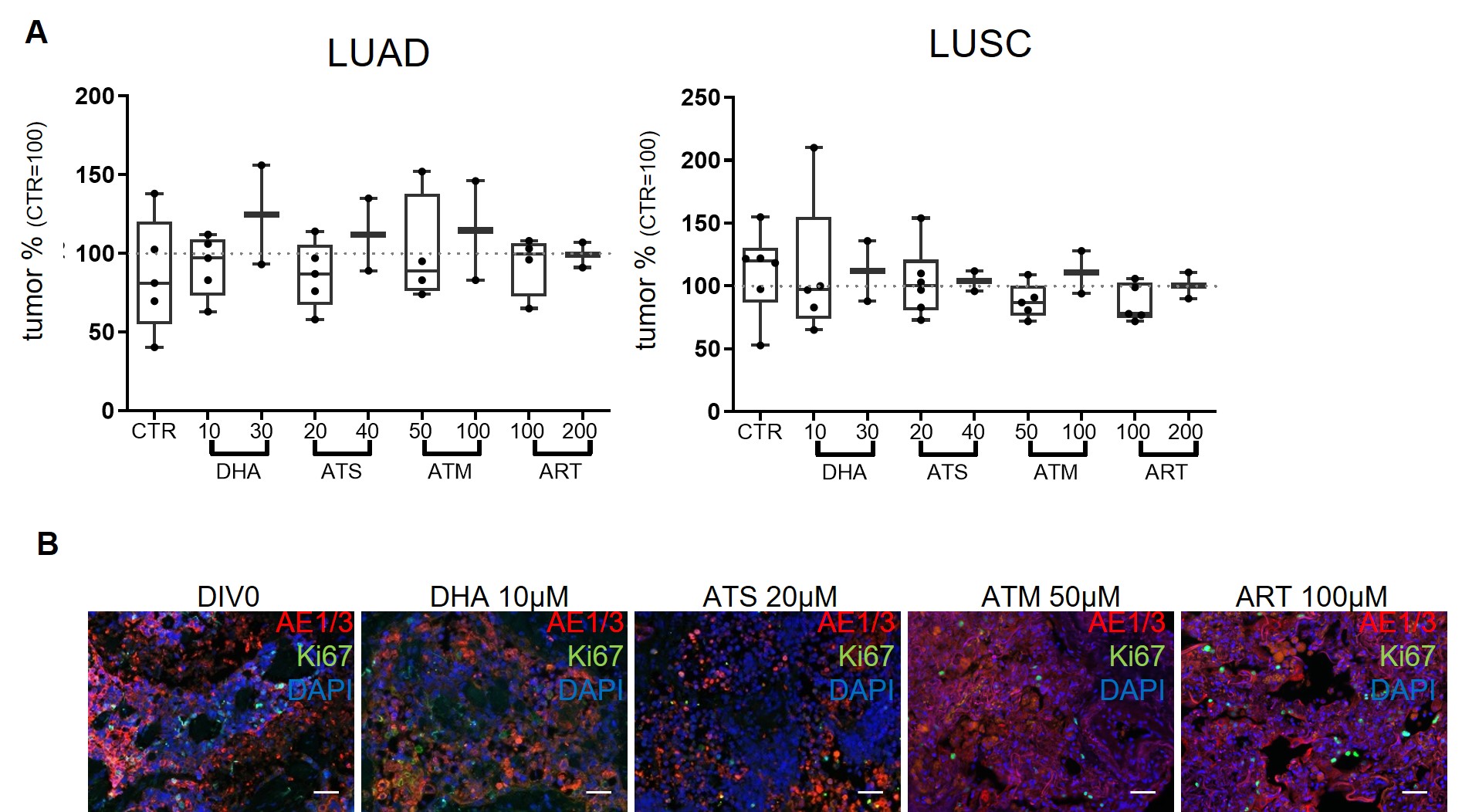

Supplement: Supplementary file 1 — Subtype dependent tumor fraction of all PDTC samples [file 41420_2025_2537_MOESM1_ESM.jpg]

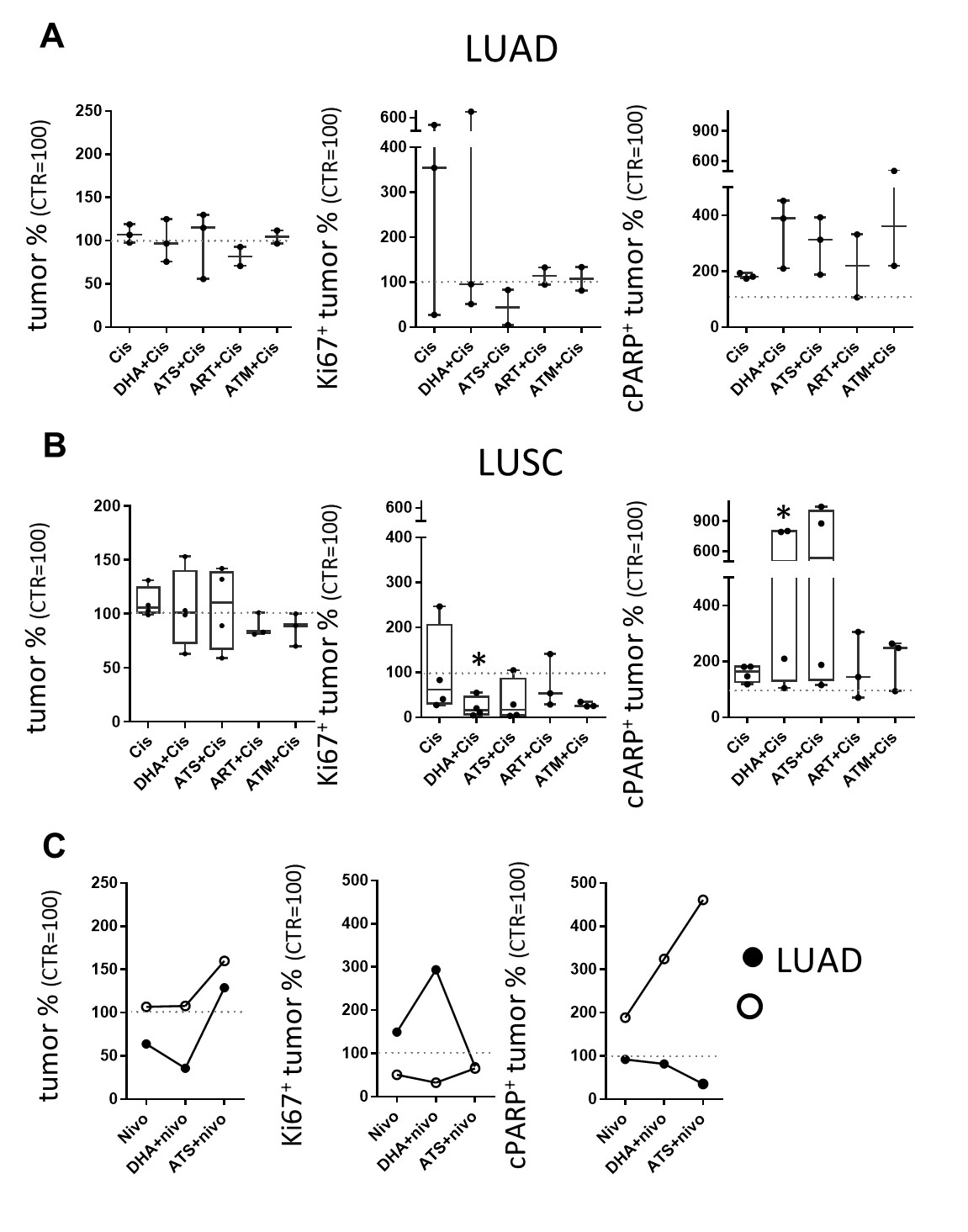

Supplement: Supplementary file 2 — Effects of cisplatin and nivolumab with artemisinin and its derivatives DHA, ATS and ATM in PDTC in NSCLC [file 41420_2025_2537_MOESM2_ESM.jpg]

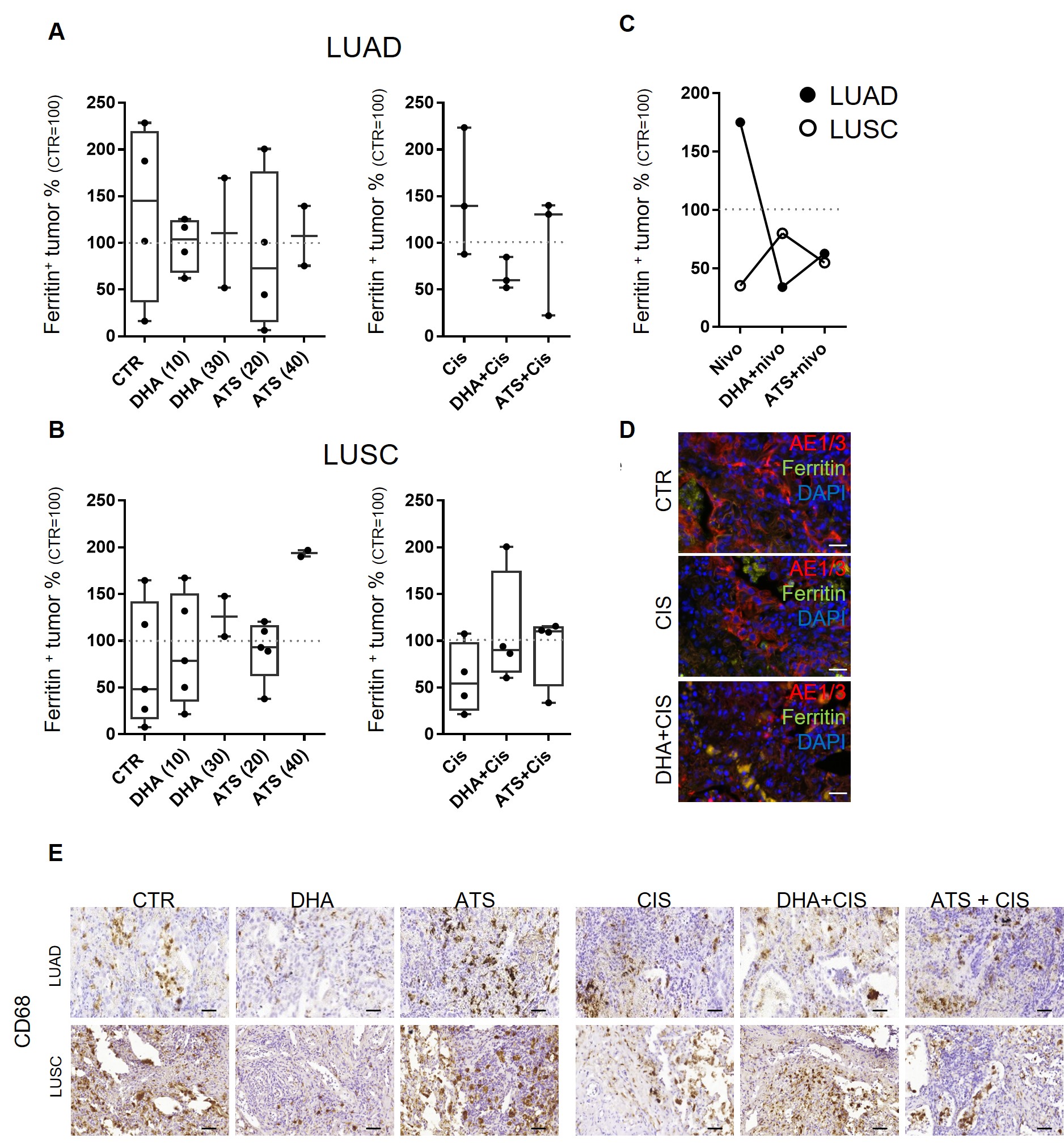

Supplement: Supplementary file 3 — Ferritin expression is not regulated by DHA and ATS in PDTC of NSCLC [file 41420_2025_2537_MOESM3_ESM.jpg]

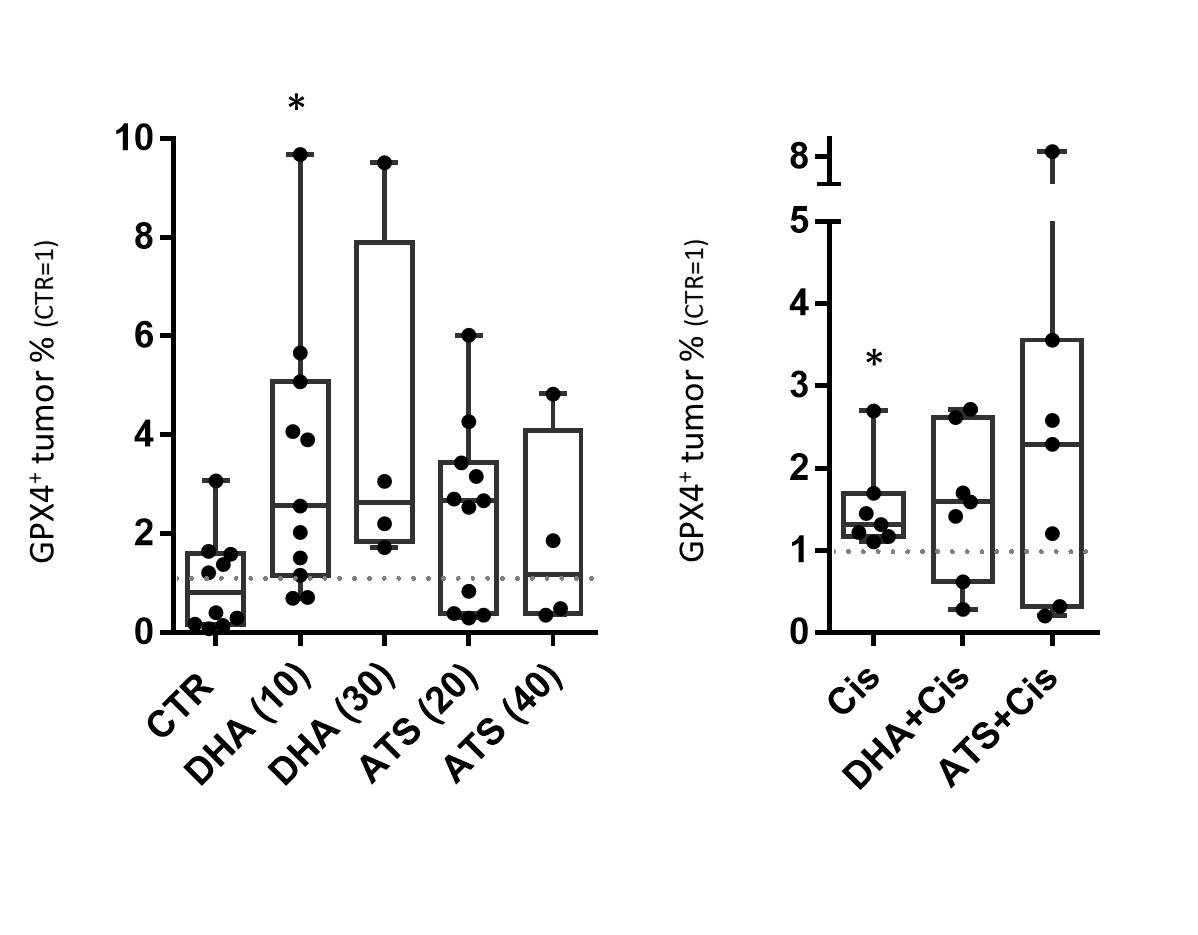

Supplement: Supplementary file 4 — GPX4 expression in PDTC of NSCLC [file 41420_2025_2537_MOESM4_ESM.jpg]
